# Comprehensive Molecular Analyses of a Novel Mutational Signature Classification System with Regard to Prognosis, Genomic Alterations, and Immune Landscape in Glioma

**DOI:** 10.3389/fmolb.2021.682084

**Published:** 2021-07-07

**Authors:** Zaoqu Liu, Taoyuan Lu, Libo Wang, Long Liu, Lifeng Li, Xinwei Han

**Affiliations:** ^1^ Department of Interventional Radiology, The First Affiliated Hospital of Zhengzhou University, Zhengzhou, China; ^2^ Interventional Institute of Zhengzhou University, Zhengzhou, China; ^3^ Interventional Treatment and Clinical Research Center of Henan Province, Zhengzhou, China; ^4^ Department of Cerebrovascular Disease, Zhengzhou University People’s Hospital, Zhengzhou, China; ^5^ Department of Hepatobiliary and Pancreatic Surgery, The First Affiliated Hospital of Zhengzhou University, Zhengzhou, China; ^6^ Internet Medical and System Applications of National Engineering Laboratory, Zhengzhou, China

**Keywords:** glioma, molecular subtype, prognosis, immunotherapy, mutational signature

## Abstract

**Background:** Glioma is the most common malignant brain tumor with complex carcinogenic process and poor prognosis. The current molecular classification cannot fully elucidate the molecular diversity of glioma.

**Methods:** Using broad public datasets, we performed cluster analysis based on the mutational signatures and further investigated the multidimensional heterogeneity of the novel glioma molecular subtypes. The clinical significance and immune landscape of four clusters also investigated. The nomogram was developed using the mutational clusters and clinical characteristics.

**Results:** Four heterogenous clusters were identified, termed C1, C2, C3, and C4, respectively. These clusters presented distinct molecular features: C1 was characterized by signature 1, PTEN mutation, chromosome seven amplification and chromosome 10 deletion; C2 was characterized by signature 8 and FLG mutation; C3 was characterized by signature 3 and 13, ATRX and TP53 mutations, and 11p15.1, 11p15.5, and 13q14.2 deletions; and C4 was characterized by signature 16, IDH1 mutation and chromosome 1p and 19q deletions. These clusters also varied in biological functions and immune status. We underlined the potential immune escape mechanisms: abundant stromal and immunosuppressive cells infiltration and immune checkpoints (ICPs) blockade in C1; lack of immune cells, low immunogenicity and antigen presentation defect in C2 and C4; and ICPs blockade in C3. Moreover, C4 possessed a better prognosis, and C1 and C3 were more likely to benefit from immunotherapy. A nomogram with excellent performance was also developed for assessing the prognosis of patients with glioma.

**Conclusion:** Our results can enhance the mastery of molecular features and facilitate the precise treatment and clinical management of glioma.

## Introduction

For Glioma is the most common malignant brain tumor associated with high heterogeneity and poor prognosis (Louis et al., 2016). The standardization regimen consisting of surgery resection combined with chemoradiotherapy is currently the dominant treatment for glioma. However, the overall therapeutic benefits remain unsatisfactory, especially glioblastoma, with a median survival of only 14.6 months after standardization therapy (Stupp et al., 2005). Hence, it is imperative to pursue new means to improve the treatment and management of glioma.

With the rapid development of bioinformatics and the rise of molecular diagnosis, precision therapy and immunotherapy have made it possible to emerge from the current dilemma (Reifenberger et al., 2017; Fecci and Sampson, 2019). The 2016 World Health Organization Classification of Tumors of the Central Nervous System incorporated molecular parameters into traditional histological diagnosis of glioma, dividing gliomas into distinct molecular phenotypes, such as IDH mutant and IDH wild gliomas, 1p/19q co-deletion and 1p/19q integrity gliomas (Louis et al., 2016). Accumulated evidence indicated that glioma patients with IDH mutation and those with 1p/19q co-deletion were relatively more sensitive to radiotherapy and chemotherapy, as well as had a favorable prognosis (Sabha et al., 2014). However, this classification only focuses on one or a few genomic alteration features, which lacks a global perspective, and fails to fully elucidate the high molecular heterogeneity of glioma. Therefore, a systematic exploration of genomic alterations in gliomas is necessary to reveal its molecular heterogeneity.

Over the past decade, immunotherapy has achieved great success in the treatment of tumors (Yang, 2015; Payandeh et al., 2019). In glioma, recent studies have reported that immunotherapy such as anti-PD-1 and anti-VEGFA could predominantly prolong the survival of some glioma patients, but the response population was not stable, only a subset of patients could benefit from immunotherapy (Sandmann et al., 2015; Lyon et al., 2017; Schalper et al., 2019). The immunotherapy limitation may be due to the high heterogeneity of gliomas and their complex immune escape mechanisms (Jackson et al., 2019; Wildes et al., 2020). Thus, investigating the molecular heterogeneity and potential immune escape mechanisms of gliomas may contribute to the development of immunotherapy.

Cancer is a complex disease arise from the constant accumulation of genomic alterations (Stratton et al., 2009). The 30 mutational signatures described by Alexandrov et al. systematically characterized the mutation accumulation leading to tumorigenesis, and linked the mutation process to DNA damage mechanisms and clinical characteristics, providing a new opportunity for in-depth analysis and understanding of the tumor molecular features (Alexandrov et al., 2015). But so far, there was no study have systematically analyzed genomic alterations and dissected mutational signatures of glioma.

Obviously, a deeper grasp of the molecular features and more refined molecular classification are essential for the precise treatment of gliomas, and the development of bioinformatics and the accumulation of broad data make it promising. In the present study, we performed molecular clustering based on the mutational signature profile of glioma patients, hoping to identify distinct molecular heterogeneous subtypes and better understand the biological characteristics of glioma. As a result, we successfully identified four heterogeneous subtypes with specific molecular characteristics, potential immune escape mechanism, and clinical outcomes in glioma. Combining the four subtypes and clinical features, we also constructed a nomogram with excellent performance to facilitate clinical prognosis management.

## Materials and Methods

### Data Source

The glioma data (*n* = 736) were enrolled from The Cancer Genome Atlas (TCGA) cohorts TCGA-LGG (low grade glioma) and TCGA-GBM (glioblastoma), the details of baseline information was shown in Supplementary Table S1. Gene expression data and clinical information were retrieved from TCGA data portal (https://portal.gdc.cancer.gov/). The mutation data, copy number alteration data, and methylation 450K data were acquired from TCGA database. Three independent immunotherapeutic cohorts containing expression and clinical data were collected: (Louis et al., 2016) metastatic melanoma patients treated with cytotoxic T lymphocyte‐associated protein 4 (CTLA-4) and PD‐1 blockades (Roh cohort) (Roh et al., 2017; Stupp et al., 2005) melanoma patients treated with adoptive T cell therapy (ACT) (GSE100797) (Lauss et al., 2017; Reifenberger et al., 2017) melanoma patients treated with anti-PD-1 (GSE78220) (Hugo et al., 2016). According to the RECIST v1.1 criterion, patients with complete response (CR) or partial response (PR) and patients with stable disease (SD) or progressive disease (PD) were deemed as immunotherapy responders and nonresponders, respectively, and patients who were not evaluable (NE) were removed.

### Identification of Mutational Signature Relevant Clusters

Somatic mutation data from TCGA-LGG and TCGA-GBM, removing silent mutations, were used for subsequent analysis. Mutational signatures described by Alexandrov et al. could be obtained from COSMIC website (Alexandrov et al., 2015). We calculated the proportion of 30 mutational signatures for each patient *via* DeconstructSigs package with signature cutoff = 0.06 and “exome2genome” normalization (Rosenthal et al., 2016). Next, the non-negative matrix factorization (NMF) algorithm was employed to perform consensus clustering and feature extraction in this study. Based on mutational signatures, consensus NMF clustering was performed *via* NMF package (Gaujoux and Seoighe, 2010) with the following parameters: potential ranks = 2–5, number of runs to perform = 50, method = “lee”. Ultimately, the optimal rank = 4 was determined by cophenetic coefficient (Supplementary Figure S1I). The “nmf” and “extractFeatures” function implemented in NMF package were utilized to extract the basis-specific features of each basis component. Out of 30 mutational signatures, 11 signatures were identified in above analysis based on method = “max” from Carmona-Saez et al. (2006). To investigate the importance of these signatures for each clinical characteristic, the decision tree C5.0 algorithm was performed with the C50 package, iterating 10 times. In addition, the APOBEC enrichment analysis described by Roberts et al. was further performed by the Maftool package (Mayakonda et al., 2018).

### Genomic Alterations Analysis

The prediction of driver genes was performed by the OncodriveCLUST, an algorithm to identify candidate driver genes with a significant bias towards mutation clustering within the protein sequence (Tamborero et al., 2013). Genes with q values less than 0.05 and mutation frequency greater than 2% or genes with mutation frequency greater than 5% were considered as drivers in this study. The GISTIC2.0 (Mermel et al., 2011) was applied to examine recurrently amplified and deleted regions, and the regions altered in greater than 15% of the samples were included in further analysis.

### Functional Annotation and Immune Infiltration Assessment

To investigate the biological behaviors among the four clusters, the gene set enrichment analysis (GSEA) was conducted based on the Hallmark gene sets (“h.all.v7.1. symbols.gmt”), and the biological function with FDR <0.05 was significance. We also explored the correlation between clusters and other related biological processes, using the gene sets proposed by Mariathasan et al. (2018) (Supplementary Table S2). Single sample gene set enrichment analysis (ssGSEA) algorithm implemented in GSVA package was applied to estimate the relative infiltration abundance of tumor microenvironment (TME) cells. The gene sets for marking 28 immune cell types were enrolled from Charoentong et al. (2017) (Supplementary Table S3). As endothelial cell and fibroblasts were also critical components of TME, we included another 40 genes from MCP-Counter gene list to mark these two cell types (Becht et al., 2016) (Supplementary Table S3).

### Collection and Investigation of Immune Escape Indicators

A series of tumor immune-related indicators (Supplementary Table S4), including stromal and leukocyte fractions, nonsilent mutation rate, neoantigen burden, cancer testis antigens (CTA) score, aneuploidy score, intratumor heterogeneity, number of segments (Segs), number or fraction of segments with loss of heterozygosity (LOH), fraction altered, homologous recombination deficiency (HRD), TCR diversity (Shannon Entropy and Richness) score (Thorsson et al., 2018), microsatellite instability (MSI) score (Bonneville et al., 2017), cytolytic activity (Rooney et al., 2015), antigen processing and presenting machinery score (APS) (Wang et al., 2019) and the expression of immunomodulator molecules (Thorsson et al., 2018), were enrolled or calculated for the investigation of potential immune escape mechanisms in the four clusters. Moreover, multi-omics regulation of 75 immunomodulator molecules was further analyzed (Supplementary Table S5), including somatic mutation, copy number variation (CNV) and DNA methylation.

### Clinical Relevance of the Four Clusters

Univariate and multivariate Cox regression analysis were performed to assess the prognostic significance of clusters and other vital clinical features. We included the features with multivariate Cox *p* value < 0.05 to construct a nomogram and further evaluated its performance by the calibration and receiver operating characteristic (ROC) curves. Subsequently, the package pRRophetic, which can predict the patients’ chemotherapeutic response based on a ridge regression (Geeleher et al., 2014), was employed to estimate the sensitivity of the four clusters to gemcitabine and bortezomib. Drug sensitivity was quantified by half-maximal inhibitory concentration (IC50), the lower the IC50, the more sensitive. Two methods were further employed to predict the immunotherapeutic response of the four clusters. First, the Tumor Immune Dysfunction and Exclusion (TIDE) algorithm was utilized to predict the immunotherapeutic response of each patient (Jiang et al., 2018). Second, subclass mapping (Hoshida et al., 2007) was applied to assess the similarity of gene expression profiles between the four clusters and three published immunotherapy cohorts (GSE100797, GSE78220, and Roh cohorts), if a pair's expression profiles shared the more similarity, their clinical outcomes were more likely to be similar.

### Statistical Analysis

The co-occurrence or exclusion of driver mutations were evaluated by Fisher exact test. Spearman correlation analyses were applied to compute the correlation coefficients of two variables. The comparisons of two groups were conducted by Wilcoxon rank-sum test, and when three or more groups, Kruskal–Wallis test was employed. The Kaplan–Meier method was applied to generate survival curves for prognosis analyses, and the log-rank test was used to define the significance of differences. The hazard ratios for variables were calculated by univariate Cox regression analyses, and multivariate Cox regression was employed to ascertain independent prognostic factors. The receiver operating characteristic (ROC) curves for survival variables were plotted by the timeROC R package. All heatmap in this study were plotted by the ComplexHeatmap package (Gu et al., 2016). All statistical *p* values were two-sided, and *p* < 0.05 was deemed as statistically significance. P-adjust value was obtained by Benjamini-Hochberg (BH) multiple test correction. All data processing was completed in R 3.6.3 software.

## Results

### Identification of Mutational Signatures Related Clusters

**FIGURE 1:**
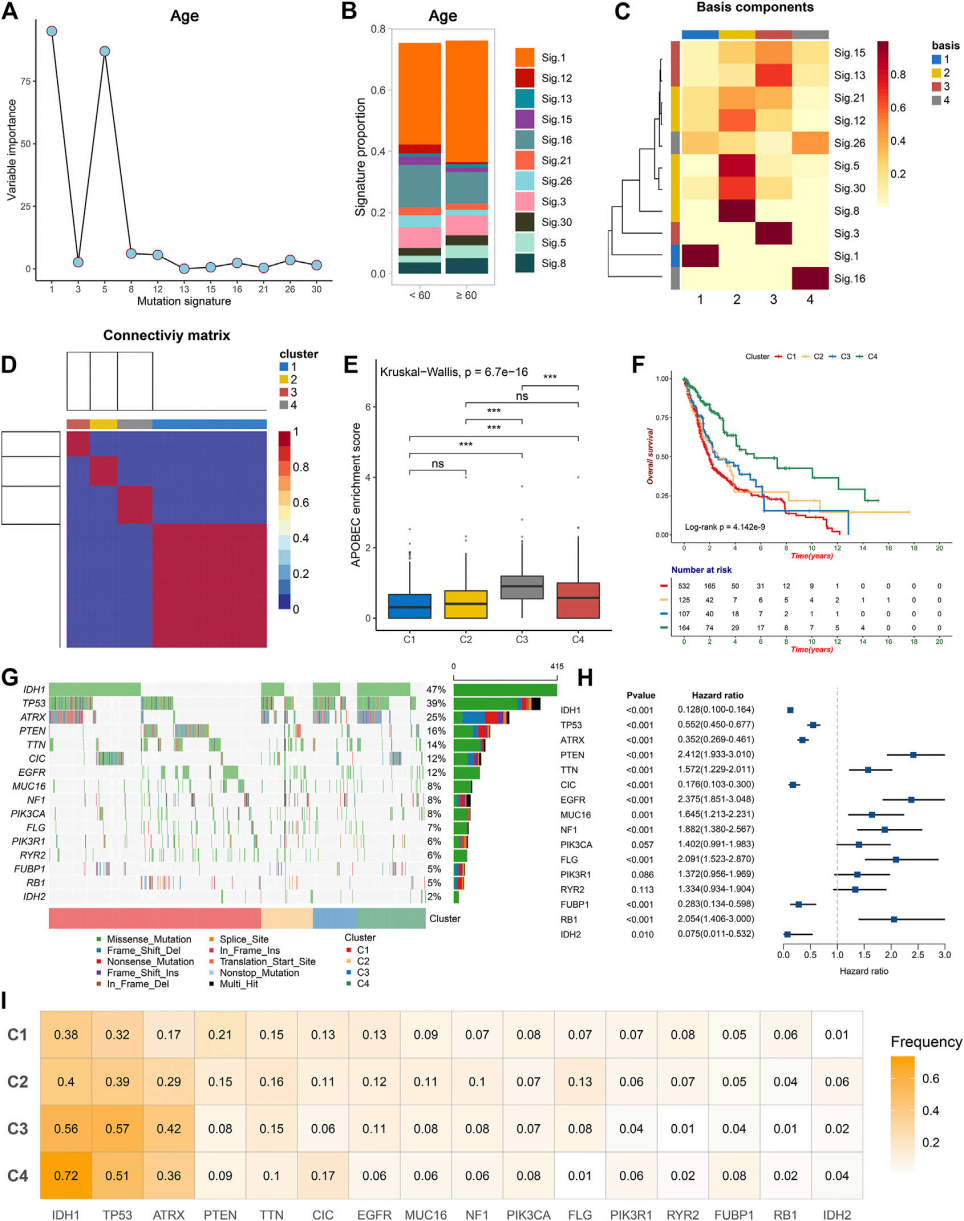
The mutational signatures and driver genes of four clusters. **(A)** Importance of 11 extracted mutational signatures in distinguishing patients of different age groups. **(B)** Distribution of 11 signatures in different age groups. **(C)** Basis component of NMF with rank = 4 in TCGA glioma cohort. **(D)** Consensus matrix after clustering revealed four clusters with no overlap between clusters. **(E)** The distribution of APOBEC enrichment score among the four clusters. The asterisks represent the statistical *p* value (ns, *p* > 0.05; ***p < 0.001). **(F)** Kaplan–Meier curves for OS among the four clusters in TCGA glioma cohort **(G)** Mutation landscape of 16 candidate driver genes in the four clusters. **(H)** Univariate Cox regression analysis of 16 candidate driver genes. **(I)** Mutation frequency of 16 candidate driver genes in the four clusters.

The somatic mutation landscape of 892 glioma patients was summarized in this study (Supplementary Figure S1A). A total of 56,369 somatic mutations were detected, and missense mutation occupied the dominant fraction. In single nucleotide variation (SNV), C > T displayed the highest frequency followed by T > C and C > A, which was consistent with the result of transition (Ti) and transversion (Tv) (Supplementary Figure S1B). In addition, we observed 14,852 mutant genes in total, and 26 genes with a mutation frequency of more than 3%. To better comprehend the contribution of these mutations to glioma, an in-depth exploration based on mutational signatures was conducted. Using the decision tree algorithm, we evaluated the importance of 11 extracted mutational signatures for various clinical characteristics in glioma (Figures 1A,B, Supplementary Figures S1C–H). There was no gender related mutational signature. Signature 1 and signature 5 were previously considered to correlate with age of cancer diagnosis, consistent with our findings in glioma (Figures 1A,B). We also observed that signature 1 was significantly associated with IDH mutation, TERT promoter mutation, MGMT promoter methylation, and the combination of chromosome seven gain and 10 loss (7+/10−). Based on previous studies, signature 3 was associated with homologous recombination deficiency (HRD), signature 13 was associated with the activation of apolipoprotein B mRNA editing enzyme, catalytic polypeptide-like (APOBEC), and signature 16 was related to alcohol and transcription-coupled damage (Letouzé et al., 2017). In this study, we also found that signature 3 was linked to 7+/10−, signature 13 was linked to ATRX mutation and MGMT promoter methylation, and signature 16 was linked to grade in glioma. The proposed etiology of signature 8 was unknown yet, but we detected it was related to MGMT promoter methylation (Supplementary Figures S1C–H). These results suggested that different mutational signatures may be associated with specific etiology and clinical characteristics in glioma.

Consensus NMF clustering was further performed to identify heterogeneity clusters based on the fraction of 11 mutational signatures, and eventually four clusters were determined, termed as C1, C2, C3, and C4 (Figures 1C,D). C1 was characterized by signature 1, which was initiated by spontaneous deamination of 5-methylcytosine and correlated with age. C2 was characterized by signature 8, which associated with MGMT promoter methylation. C3 was characterized by signature 3 and signature 13, linked to HRD and the activation of APOBEC. The APOBEC enrichment results further revealed that C3 exhibited a higher score (Figure 1E). C4 was characterized by signature 16 associated with drinking and transcription-coupled damage (Figure 1C). Survival analysis revealed the different prognosis of four clusters, and C4 exhibited a favorable prognosis (Figure 1F).

### Mutation Driver Genes

The tumor mutation burden (TMB) investigation displayed a decreasing trend from C1 to C4, although the difference between four clusters was not very pronounced (Supplementary Figure S2A). A total of 16 candidate driver genes were identified (Supplementary Table S6; Supplementary Figure S2B; Figure 1G). It was noted that there was a significant mutation co-occurrence between IDH1, ATRX, and TP53, which often appeared in astrocytoma. We also observed an exclusion between IDH1 and EGFR, IDH1 and PTEN, along with a co-occurrence between EGFR and PTEN, which often appeared in glioblastoma (Supplementary Figure S2C) (Diplas et al., 2018). We visualized the hotspots and other mutations in glioma *via* lollipop plots (Supplementary Figure S2D). For example, the IDH1 mutation focused on residue 132 of PTZ00435, consistent with previous cognition (Kloosterhof et al., 2011). Univariate cox regression and survival analysis further revealed the prognostic value of these 16 driver genes (Figure 1H; Supplementary Figure S2E). Out of these genes, IDH1, IDH2, TP53, ATRX, CIC, and FUBP1 mutations were favorable factors for prognosis, while others were poor factors. In addition, we also investigated the mutation frequency of the driver genes in each cluster. It was found that C1 was characterized by PTEN, C2 was characterized by FLG, C3 was characterized by TP53 and ATRX, and C4 was characterized by IDH1 (Figure 1I). In C4, genes that favor prognosis exhibited a relatively higher mutation frequency, while gene mutations that disfavor prognosis had a relatively low frequency, which may constitute an explanation for the better survival status of C4.

### Significantly Altered Segments

**FIGURE 2:**
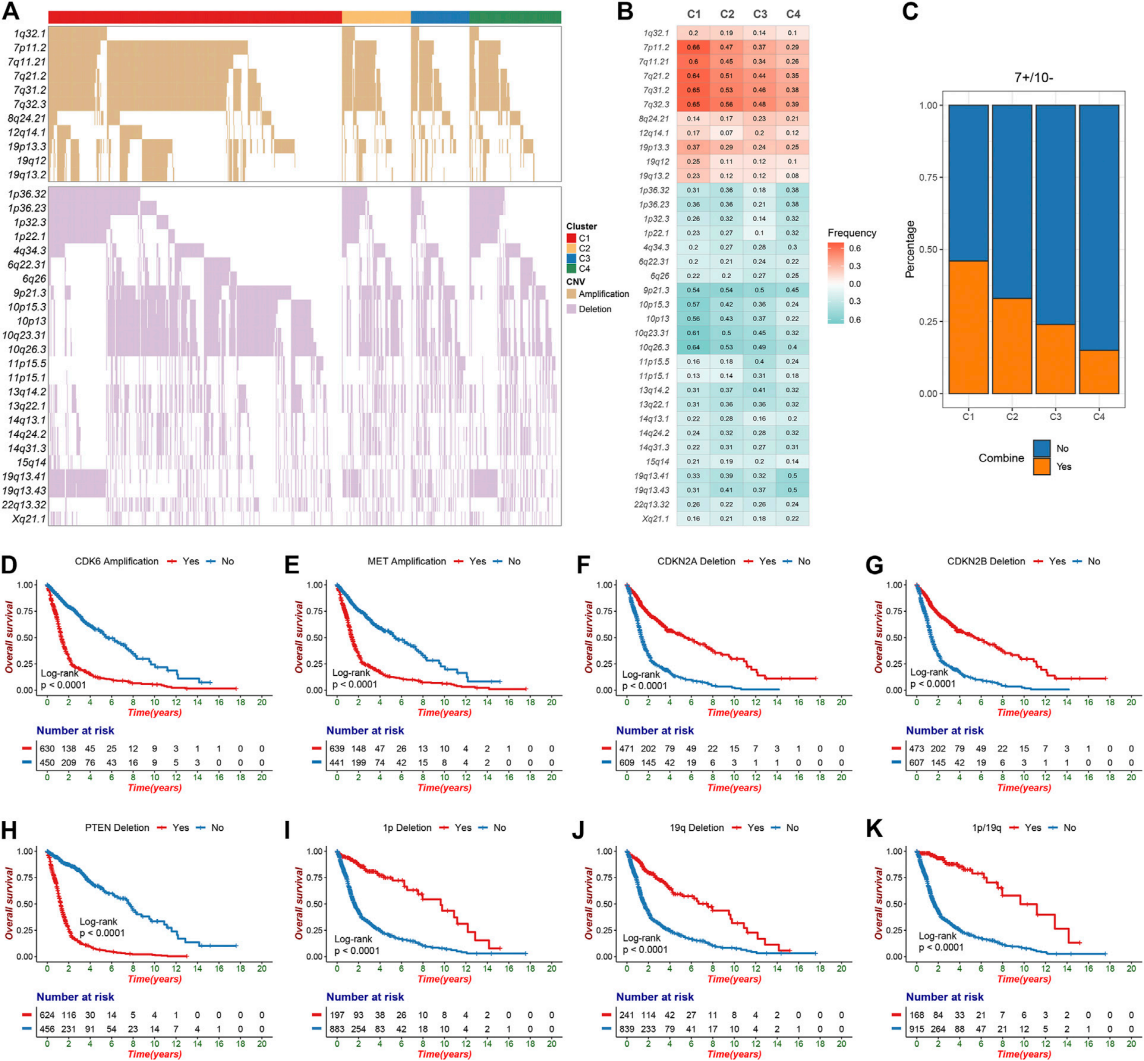
The significant recurrent segments obtained from GISTIC algorithm in TCGA glioma cohort. **(A)** The oncoplot of 35 frequently segments in the four clusters. **(B)** Frequency of amplification (red) and deletion (green) among the four clusters. **(C)** The distribution of combined chromosome seven gain and 10 loss in the four clusters. (**D**–**H)** Kaplan-Meier survival analysis of CDK6 **(D)** and MET **(E)** amplifications as well as CDKN2A (**F**), CDKN2B **(G)** and PTEN **(H)** deletions. **(I**-**K)** Chromosome 1p deletion **(I)**, chromosome 19q deletion **(J)** or 1p/19q co-deletion **(K)** were associated with poor overall survival in glioma patients.

The GISTIC algorithm revealed the landscape of significantly recurrent amplification and deletion in glioma (Supplementary Tables S7,S8; Supplementary Figures S3A,B). A total of 35 segments with alteration frequency more than 15% were selected for further analysis (Figure 2A), and univariate Cox regression assessed the prognostic significance of these segments (Supplementary Figure S3C). Of these, we revealed for the first time that in glioma, gains of 12q14.1 and losses of 6q22.31, 6q26, 13q14.2, 13q22.1, 15q14, and 22q13.32 were significantly linked to a poor prognosis whereas loss of 4q34.3 was linked to a favorable prognosis. C1 was characterized by the most frequent alterations encompassing five amplifications on chromosome seven and four deletions on chromosome 10, all linked to poor prognosis (Figures 2B,C; Supplementary Figure S3C). Oncogenes such as CDK6 (7q21.2) and MET (7q31.2) were appreciably amplified, whereas tumor suppressor genes (TSGs) such as CDKN2A/2B (9p21.3) and PTEN (10q23.31) were significantly deleted, which may contribute to poor outcomes in patients with chromosome seven gain, chromosome 9p loss or chromosome 10 loss (Supplementary Tables S7,S8; Figures 2D–H; Supplementary Figures S3D–G). The combined chromosome seven gain and chromosome 10 loss (7+/10−) was altered frequently in some gliomas (Stichel et al., 2018), we thus investigated the incidence of 7+/10− among four clusters and found that it was highest in C1 (*p* = 2.33e-12) (Figure 2C). C3 exhibited higher frequency of 11p15.1, 11p15.5, and 13q14.2 deletions in contrast to the other clusters. Four deletions on 1p and two on 19q which linked to a favorable prognosis were most frequently altered in C4 (Figure 2B; Supplementary Figure S3C). Of note, patients with TSG-associated deletions including 1p32.3 (CDKN2C), 1p36.23 (ERRFI1), 1p36.32 (AJAP1 and HES3), and 19q13.41 (PPP2R1A) had a survival advantage over no deletions (Figures 2I–K; Supplementary Table S8). This can be explained by the fact that 1p/19q co-deletion was a driving event in oligodendroglioma but could increase patient sensitivity to chemoradiotherapy (Kaloshi et al., 2007; Barthel et al., 2018). In addition, another deletion significantly associated with a favorable prognosis, 4q34.3, also had the highest alteration frequency in C4.

### Biological Characteristics of the Four Clusters

**FIGURE 3:**
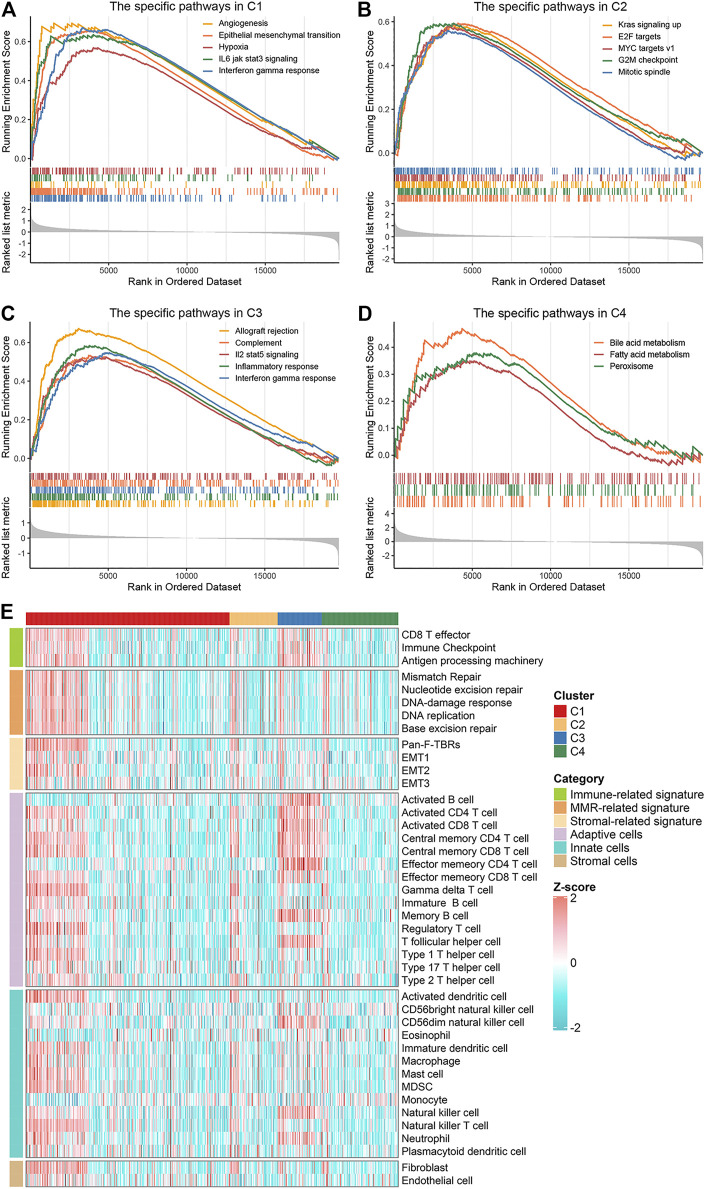
Biological characteristics of the four clusters. **(A–D)** GSEA enrichment analysis revealed activated Hallmark pathways of C1 **(A)**, C2 **(B)**, C3 **(C)** and C4 **(D)**, the FDR of the biological function was <0.05. **(E)** The distribution of known signatures (immune-relevant signatures, mismatch-relevant signatures, and stromal-relevant signatures) and TME cells assessment (adaptive immune cells, innate immune cells and stromal cells) in the four clusters.

To explore and characterize the biological behaviors among the four clusters, we performed GSEA enrichment analysis. C1 was enriched in stromal and immune activation relevant pathways, such as angiogenesis, epithelial mesenchymal transition, hypoxia, IL-6 JAK STAT3 signaling, and IFN-γ response (Figure 3A). C2 presented pathways involved in promoting proliferation such as KRAS signaling, E2F targets and G2M checkpoint (Figure 3B). C3 displayed intense pathways associated with immune activation encompassing allograft rejection, complement, IL-2 STAT5 signaling, inflammatory response, and IFN-γ response (Figure 3C). C4 was enriched in pathways pertinent to metabolism such as bile acid metabolism, fatty acid metabolism, and peroxisome (Figure 3D). We also examined known signatures in four clusters to better understand the functionality of them. Stroma-related signatures such as epithelial-mesenchymal transition (EMT) and pan-fibroblast TGF beta response (Pan-F-TBR), and mismatch repair (MMR)-related signatures such as base excision repair, were markedly enhanced in C1; immune-related signatures such as antigen processing machinery, CD8 T effector and immune-checkpoint were appreciably enhanced in C3 (Figure 3E, Supplementary Figure S4A). This confirmed the findings in GSEA. Of note, C1 exhibited not only stromal activation but also immune activation. Analysis of TME cell infiltration demonstrated that immune cells such as activated CD4^+^ T cell and activated CD8^+^ T cell were most abundant in C3, followed by C1; stromal activation-associated cells such as endothelial cell and fibroblasts were most enriched in C1 (Figure 3E, Supplementary Figure S4B). This confirmed again that C1 was characterized by stromal and immune dual activation, while C3 was characterized by immune activation. Moreover, C1 also displayed the highest infiltration of MDSC and regulatory T cell (Figure 3E, Supplementary Figure S4B). We speculated that with the stromal activation, C1 may progress from an immune activation state similar to C3, towards an immunosuppressive state. Overall, C1 was classified as stromal and immune dual activation phenotype, C2 was classified as proliferation phenotype, C3 was classified as immune activation phenotype, and C4 was classified as metabolism phenotype.

### Potential Extrinsic Immune Escape mechanisms

**FIGURE 4:**
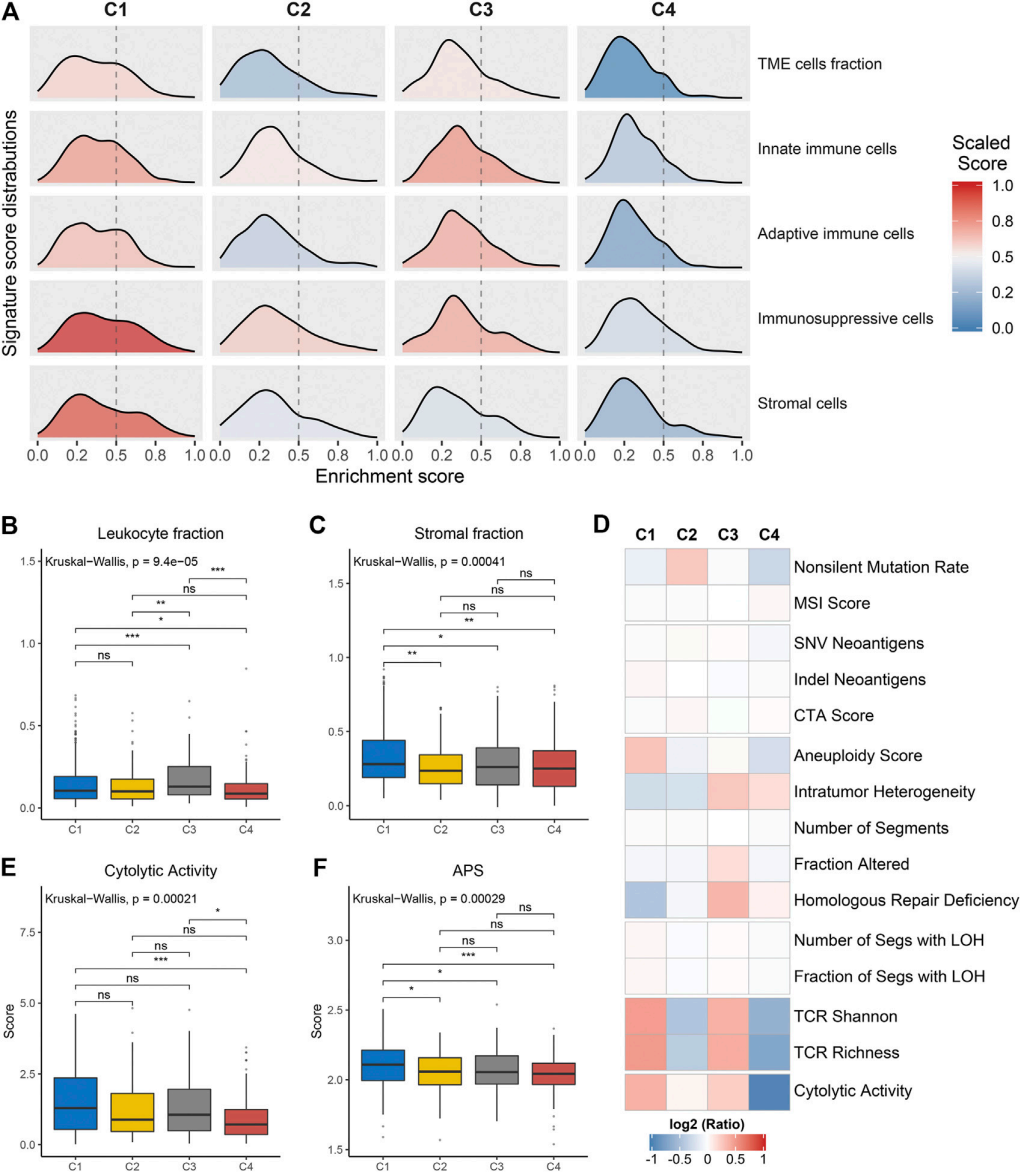
Potential extrinsic immune escape mechanisms of each cluster. **(A)** The scaled signature score distributions of five cell subsets among the four clusters. **(B,C)** Comparison of leukocyte fraction **(B)** and stromal fraction **(C)** among the four clusters. **(D)** Comparison of 14 immunogenicity associated indicators among the four clusters, the cell represented by the mean value of corresponding cluster divided by the overall mean value. **(E,F)** Comparison of cytolytic activity **(E)** and APS **(F)** among the four clusters. For all boxplots, the asterisks represent the statistical *p* value (ns, *p* > 0.05; *p < 0.05; **p < 0.01; ***p < 0.001).

We further investigated the specific immune escape mechanisms of each subtype. Extrinsic immune escape mainly encompassed absence of immune cells, emergence of immunosuppressive cells, and high abundance of stromal cells (Schumacher and Schreiber, 2015; Wang et al., 2019). We pooled together the relative abundance of TME cells among four clusters. C1 and C3 exhibited a high level of TME cells, innate immune cells and adaptive immune cells, which were considered as immune-hot phenotypes. Whereas C2 and C4 demonstrated overall low TME cell levels, which were considered as immune-cold phenotypes (Figure 4A, Supplementary Figures S5A–C). In addition, C1 also displayed significantly superior levels of immunosuppressive cells and stromal cells (Supplementary Figures S5D,E), suggesting an immune-hot but suppressive microenvironment. This phenomenon may contribute to the extrinsic immune escape of C1. The stromal and leukocyte fractions from Thorsson et al. study also indicated that C1 was characterized by high stromal fraction while C3 was characterized by high leukocyte fraction, further validating the above findings (Figures 4B,C). In term of C2 and C4, the lack of immune cells implied an inability to immunologically eliminate tumor. Molecules associated with initiation of innate immunity, such as CLEC7A, PYCARD and TLR2, were also relatively low expressed in these two clusters (Supplementary Figure S5F). These results illustrated that absence of immune cells may be an extrinsic immune escape mechanism for C2 and C4.

### Potential Intrinsic Immune Escape mechanisms

**FIGURE 5:**
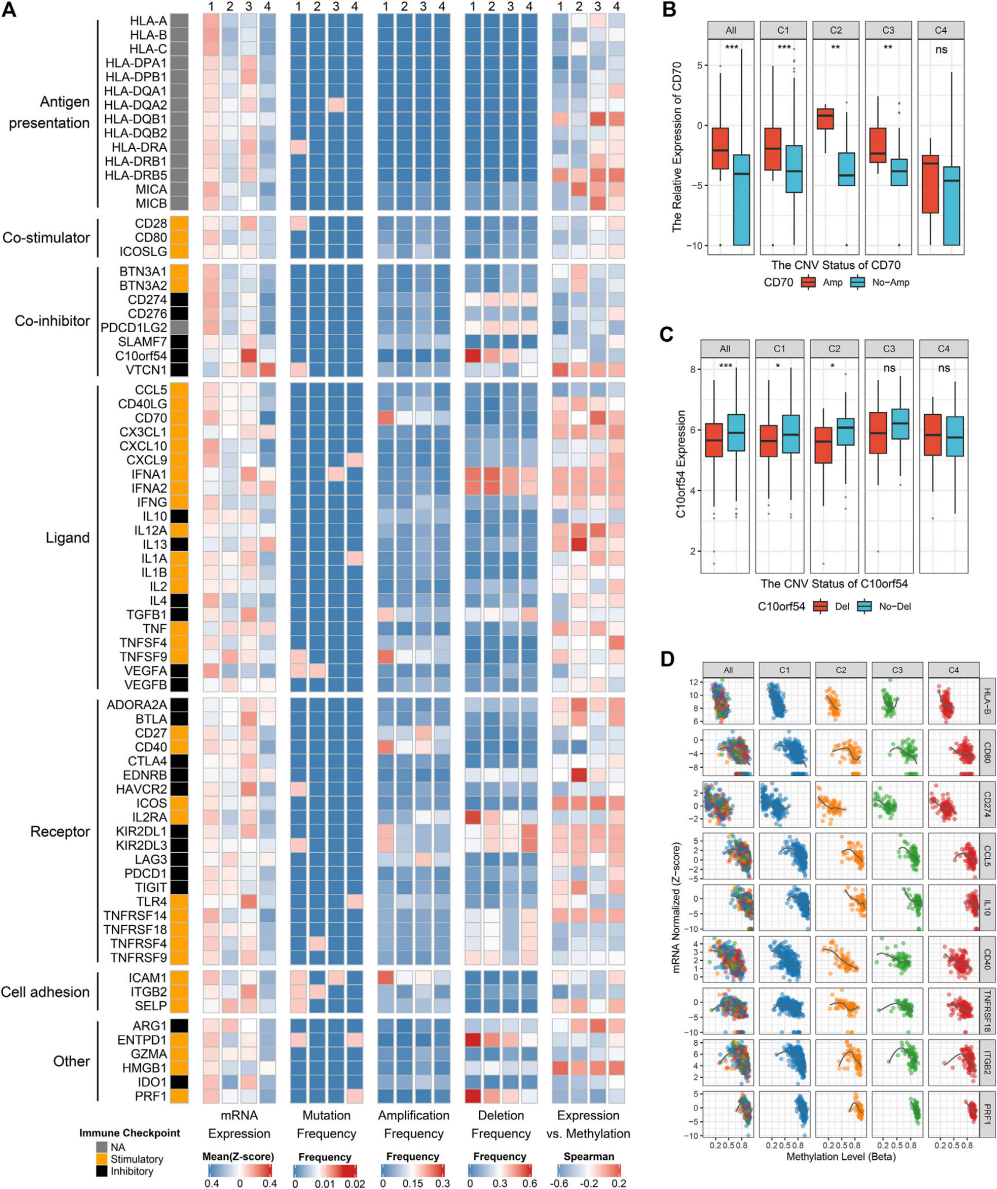
Multi-omics analysis of 75 immunomodulators in glioma. **(A)** From left to right: mRNA expression (z-score), mutation frequency, amplification frequency, deletion frequency, and expression vs. methylation (gene expression correlation with DNA-methylation beta-value) of 75 immunomodulators in the four clusters. **(B)** Comparison of CD70 relative expression between amplification and non-amplification groups. **(C)** Comparison of C10orf54 relative expression between deletion and non-deletion groups. **(D)** Correlation analysis of DNA methylation levels and mRNA expression levels for HLA-B, CD80, CD274, CCL5, IL10, CD40, TNFRSF18, ITGB2, and PRF1. For boxplot, the asterisks represent the statistical *p* value (ns, *p* > 0.05; *p < 0.05; **p < 0.01; ***p < 0.001).

The exploration of intrinsic immune escape mechanism mainly included three major aspects: tumor immunogenicity, antigen presentation capacity and immune checkpoint molecules expression (Schumacher and Schreiber, 2015). We first assessed a series of factors associated with tumor antigenicity, including mutations, MSI, neoantigens, CTA, and CNV-related indicators (Figure 4D). C4 exhibited a lower rate of nonsilent mutation compared to C1 and C2 (*p* < 0.05; Figure 4D, Supplementary Figure S6A). MSI score displayed a decreasing trend from C4 to C1, although it was not significant (Figure 4D, Supplementary Figure S6B). Neoantigens and CTA were also vital source of tumor-specific antigens, but they were not significantly different between the four clusters (Figure 4D, Supplementary Figures S6C–E). C1 presented higher aneuploidy score and fraction of segments with LOH, in contrast to the other three clusters (*p* < 0.05; Figure 4D, Supplementary Figure S6F, Supplementary Figure S6L). C3 exhibited a high level of homologous recombination deficiency, consistent with its mutation cluster characteristics (mutational signature 3) (Figure 4D, Supplementary Figure S6J). In addition, TCR diversity and cytolytic activity were applied to further assess tumor immunogenicity (Rooney et al., 2015). C2 and C4 exhibited a lack of TCR diversity and low cytolytic activity, as opposed to C1 and C3 (Figures 4D,E, Supplementary Figures S6M,N). Overall, C2 and C4 displayed lower immunogenicity, which may be an intrinsic immune escape mechanism for these two clusters. In term of antigen processing and presenting machinery, C1 exhibited the highest APS while C2 and C4 were quite the opposite (*p* < 0.05; Figure 4F). Expression of MHC molecular were also relatively low in C2 and C4 (Figure 5A). Of note, corresponding to MHC loss, TCR diversity was also lacking in C2 and C4. The absence of MHC stimulation may be responsible for the scarcity of TCR diversity in these two clusters (Figure 4D). Therefore, we believed the defect of antigen presentation capacity may be another intrinsic immune escape mechanism for C2 and C4.

### Multi-Omics Analysis of Immunomodulators

The expression and regulation of immune checkpoint molecules were also a crucial intrinsic immune escape mechanism (Figure 5A). In this research, the expression of immunomodulators varied across the clusters, with the vast majority being highly expressed in C1 and C3, but quite low in C4 (Figure 5A). C1 had higher expression of many stimulators (e.g., CD80, CCL5, CD70, and PRF1) and inhibitors (e.g., CD274 and VEGFA) compared with the other three clusters. C3 exhibited markedly high expression of stimulators such as TLR4, and inhibitors such as C10orf54, CTLA4 and HAVCR2. These results hinted that C1 and C3 may escape immune elimination by overexpressing immune inhibitors after stimulating immune activation, implying an intrinsic immune escape mechanism for these two clusters.

To advance this investigation, we further analyzed the multi-omics features of the immunomodulators among the four clusters (Figure 5A). Most immunomodulators presented rare somatic mutations. In term of CNVs, C1 exhibited frequent amplification and deletion of many immunomodulator genes such as CD70, ICAM1, C10orf54, etc., in line with the high genomic instability of C1. Of note, we found the expression levels of CD70, CD40, and ICAM1 with amplification were higher than those without amplifications, while C10orf54 with deletion displayed lower expression level relative to no deletion (Figures 5A–C, Supplementary Figures S6O,P). This phenomenon indicated that CNVs played a non-negligible role in regulating the expression of certain immunomodulators. We also detected that DNA methylation levels of many immunomodulator genes, such as HLA-B, CD80, CD274, CCL5, IL10, CD40, TNFRSF18, ITGB2, and PRF1, were inversely correlated with their gene expression levels, implying their essential role by epigenetic silencing (Figures 5A,D). In summary, CNVs and methylation modification were prominent participants in the regulation of immunomodulators, which suggested a novel orientation for the development of immune therapy.

### Distinct Clusters Associated With Different Clinical Outcomes

**FIGURE 6:**
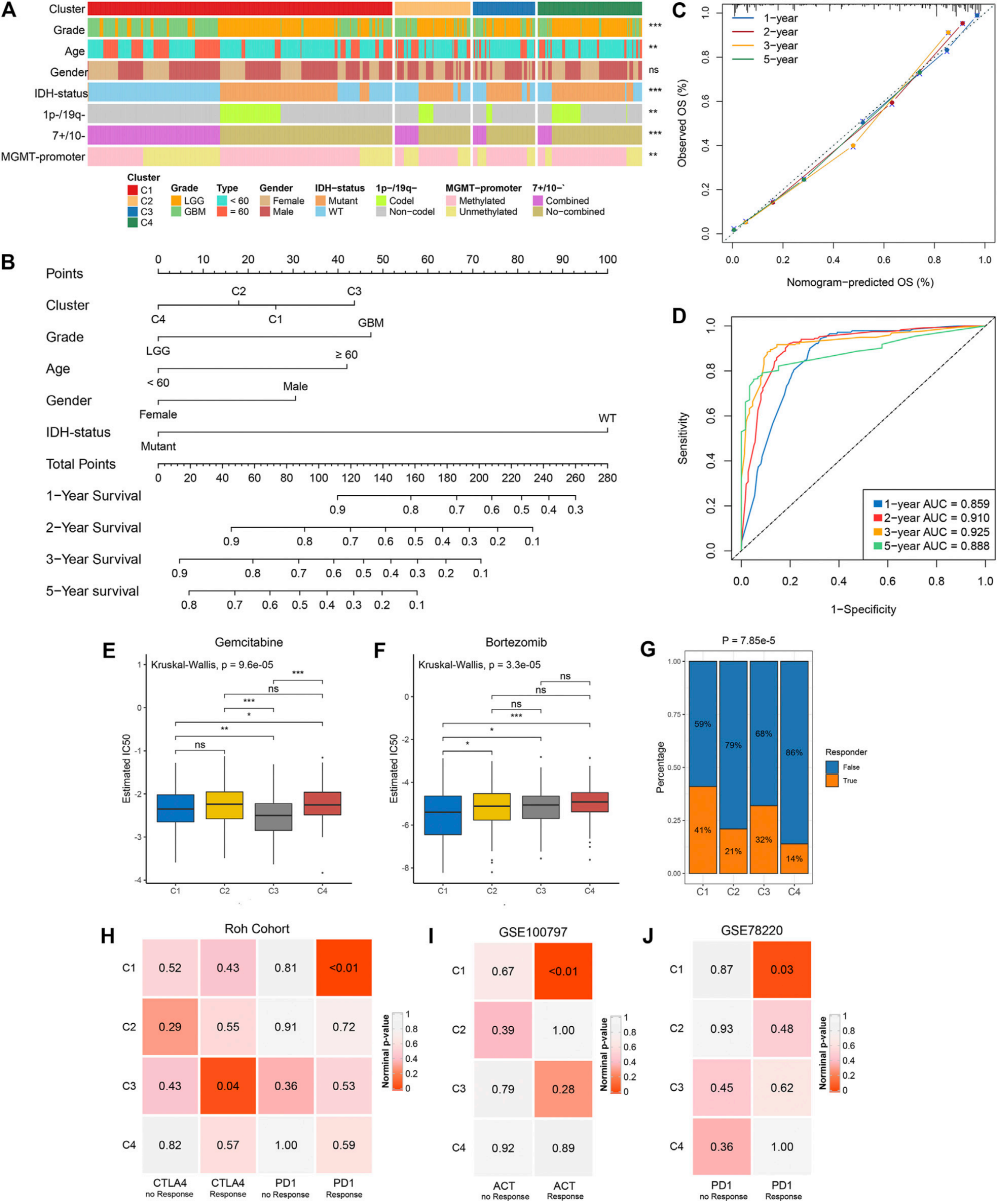
Clinical relevance of the four clusters in TCGA glioma cohort. **(A)** Distribution of grade, age, gender, IDH status, 1p-/19q−, 7+/10− and MGMT-promoter methylation in the four clusters. The right asterisks represent the statistical *p* value (ns, *p* > 0.05; **p < 0.01; ***p < 0.001) for significance of the difference among clusters. **(B)** The nomogram for predicting the 1-, 2-, 3- ,and 5-years survival possibility of individuals. **(C,D)** Calibration curve **(C)** and ROC curve **(D)** for evaluating the performance of nomogram. **(E,F)** The estimated IC50 of gemcitabine **(E)** and bortezomib **(F)** among the four clusters. The asterisks represent the statistical *p* value (ns, *p* > 0.05; *p < 0.05; **p < 0.01; ***p < 0.001). **(G)** The distribution of the immunotherapy responders predicted by TIDE algorithm in the four clusters. **(H)** Submap analysis of the four clusters and Roh cohort with detailed anti-PD1 and anti CTLA4 therapy information. **(I)** Submap analysis of the four clusters and GSE100797 with detailed ACT information. **(J)** Submap analysis of the four clusters and GSE78220 with detailed anti-PD1 therapy information. For submap analysis, a smaller *p*-value implied a more similarity of paired expression profiles.

We examined the distribution of clinical characteristics including grade, age, gender, IDH-status, 1p−/19q−, 7+/10− and MGMT-promoter methylation in the four clusters (Figure 6A). The percentage of elderly patients and senior grade glioma patients displayed a decreasing trend from C1 to C4 (Supplementary Figures S7A,B). There was no significant difference in gender distribution among the four clusters (Figure 6A; Supplementary Figure S7C). Of note, C4 had the highest percentage of IDH mutation, 1p/19q codeletion and MGMT-promoter methylation (Supplementary Figures S7D–F; Figure 2C). Univariate and multivariate Cox regression analysis further revealed the prognosis value of these characteristics, and then we identified five independent prognostic factors encompassing the clusters, grade, age, gender and IDH-status (Supplementary Table S9). Based on these five factors, we developed a nomogram to assess the 1-year, 2-years, 3-years, and 5-years survival of individual patients (Figure 6B). The calibration curve demonstrated good agreement between nomogram-predictions and observations (Figure 6C). The Area Under the Curve (AUC) of ROC curve for 1-year, 2-years, 3-years and 5-years were 0.859, 0.910, 0.925, and 0.888, respectively (Figure 6D). These results suggested that the nomogram had an excellent performance. In addition, the chemotherapy and immunotherapy sensitivity of each cluster was further evaluated. We first predicted the response of the four clusters to two chemotherapeutic drugs: gemcitabine and bortezomib, which can benefit glioma patients in combination with standard chemotherapeutic drug temozolomide (Bastiancich et al., 2018; Kong et al., 2018). In contrast to the other clusters, C1 and C3 were more sensitive to bortezomib and gemcitabine, respectively (all *p*-value <0.05) (Figures 6E,F). The previous results suggested that C1 and C3 were considered as immune-hot subtypes, while C2 and C4 were considered as immune-cold subtypes. Therefore, we further investigated the sensitivity of each cluster to immunotherapy. The TIDE algorithm was utilized to assess the immunotherapeutic response of patients in each cluster, and it indicated that C1 (41%) and C3 (32%) had a higher response rate relative to C2 (21%) and C4 (14%) (Figure 6G). Subclass mapping analysis in Roh cohort (Roh et al., 2017) revealed that C1 displayed high similarity with patients who responded to anti-PD1 therapy (*p*-value <0.01), and C3 was significantly similar with anti-CTLA4 treatment responders (*p*-value = 0.04), implying that C1 and C3 were more prospective to respond anti-PD1 and anti-CTLA4 immunotherapy, respectively (Figure 6H). This phenomenon precisely corresponded to the high expression level of CD274 (PD-L1) in C1 and CTLA4 in C3 (Supplementary Figures S7G,H). In another two cohorts, GSE100797 and GSE78220, C1 was more likely responded to adoptive cell therapy (ACT, *p*-value <0.01) and anti-PD1 treatment (*p*-value = 0.03), further demonstrating that C1 was more promising to benefit from immunotherapy (Figures 6I,J).

## Discussion

Glioma is characterized by high heterogeneity and complex immune escape mechanism, which are increasingly recognized as critical factors that limit the progress of glioma treatment (Reardon and Wen, 2015; Jackson et al., 2019). Specific genomic alterations drive the formation of multidimensional heterogeneity in gliomas (Barthel et al., 2018). Mutational signatures that characterize different mutational processes play a crucial role in the investigation of genomic variation. Our study identified four distinct clusters based on mutational signatures, evidencing the intertumoral molecular variability in glioma. These clusters varied regarding genomic variation, biological characteristics, underlying immune escape mechanisms and clinical characteristics (Supplementary Table S10). To the best of our knowledge, the present study is the first to dissect the mutational signatures of glioma, and systematically investigate molecular heterogeneity of glioma from the perspective of genomic variation and immune escape. Meanwhile, we revealed plenty of prognosis relevant genomic driver events. In addition, the nomogram was developed to serve as a robust and promising tool for predicting the prognosis of glioma patients.

Basically, the four clusters enriched in specific mutational processes with different DNA damage mechanisms. C1 was characterized by signature 1 and related to spontaneous deamination of 5-methylcytosine, which was reported to be able to mediate a high incidence of C > T transition in some tumor-suppressor genes and play a role in carcinogenesis of human tumors (Laird and Jaenisch, 1994). Signature 8 was the characteristic of C2, but its proposed etiology was unknown yet. Based on the results of decision tree analysis, we hypothesized that signature 8 was associated with MGMT promoter methylation, which can epigenetically silence the DNA mismatch repair enzyme MGMT (Hegi et al., 2005). In C3, the characteristics were signature 3, associated with homologous recombination deficiency, and signature 13, linked to APOBEC activation. Cytosine deamination of genomic DNA catalyzed by APOBEC family members is a mechanism fueling cancer heterogeneity and evolution (Swanton et al., 2015). In C4, the characteristic was signature 16, associated with alcohol consumption and transcription-coupled damage according to a recent study (Letouzé et al., 2017). DNA damage caused by various mechanisms drives genomic instability and ultimately the cancer process (Lord and Ashworth, 2012). Therefore, the distinct mutational processes may translate into different molecular and clinical features among the four clusters.

Unsurprisingly, the present study detected a significant heterogeneity among the four clusters in genomic variation. A total of 16 significantly mutated genes were identified as drivers involved in the tumorigenesis and evolution of gliomas. Of these, PTEN mutation, co-occurring with EGFR but repelling with IDH1 mutation, was more specific to C1; FLG mutation, repulsive to IDH mutation, occurred more frequently in C2; ATRX and TP53 mutations were significantly enriched in C3; and IDH1 mutation was more specific to C4. PTEN, EGFR, ATRX, TP53 and IDH mutations were all oncogenic drivers, predominantly related to molecular diagnosis and different prognosis in glioma (Louis et al., 2016; Diplas et al., 2018). FLG mutation, associated with ichthyosis vulgaris and atopic eczema (Akiyama, 2010), was first identified as a biomarker linked to a poor prognosis in glioma. For copy number variation, the clusters also exhibited distinct characteristics, as summarized in Supplementary Table S10. The most frequent alterations were located on chromosomes 1p, 7, 10, and 19q, in line with the focus of earlier studies (Louis et al., 2016; Stichel et al., 2018). Copy number variation can lead to oncogene activation or tumor suppressor gene (TSG) inactivation in cancer. In the present study, it was detected that 7q21.2 (CDK6) and 7q31.2 (MET), more specific to C1, were appreciably amplified; 10q23.31 (PTEN), occurred more frequently in C1, and 1p32.3 (CDKN2C), 1p36.23 (ERRFI1), 1p36.32 (AJAP1 and HES3), and 19q13.41 (PPP2R1A), more specific to C4, were appreciably deleted. These oncogene-relevant amplifications and TSG-relevant deletions may contribute to the tumorigenesis and progression of glioma. In addition, the present study also revealed that 12q14.1 amplification, and 6q22.31, 6q26, 13q14.2, 13q22.1, 15q14, 22q13.32, and 4q34.3 deletions were significantly related to prognosis in glioma, implying that these alterations may be able to serve as novel prognostic biomarkers.

The heterogeneity among the four clusters was also reflected in biological function and immune status. As described, C1 was characterized by activation of stroma and immunity and high infiltration of immune and stromal cells, corresponding to stromal and immune dual activation phenotype; C2 was characterized by proliferation promotion, corresponding to proliferation phenotype; C3 was characterized by activation of immunity and high immune cells infiltration, corresponding to immune activation phenotype; and C4 was characterized by metabolism-related pathways, corresponding to metabolism phenotype. Further, we summarized and underlined the potential immune escape mechanisms of each cluster: abundant stromal and immunosuppressive cells infiltration and immune checkpoint blockade in C1; lack of immune cells, low immunogenicity and antigen presentation defect in C2 and C4; and immune checkpoint blockade in C3. The comprehensive understanding of distinct biological characteristics and potential immune escape mechanisms could guide more effective personalized therapy. Moreover, we detected that CNVs and methylation modification were prominent participants in the regulation of immunomodulators, which suggested a novel orientation for the development of glioma immunotherapy.

The present study had important implications for clinical translations and application. Synchronization with heterogeneous molecular features, the four clusters also varied in clinical characteristics, as summarized in Supplementary Table S10. First, the novel molecular subtypes had prognostic significance. C4 displayed a better OS in contrast to the other clusters. Consistently, this cluster also exhibited a higher percentage of IDH mutation, 1p/19q codeletion and MGMT-promoter methylation, which were all reported being associated with a favor prognosis in glioma (Hegi et al., 2005; Sabha et al., 2014). Based on the clusters, grade, age, gender and IDH-status, an accurate nomogram was developed to predict the 1-year, 2-years, 3-years, and 5-years survival of individual patients. Second, our results can provide a reference for the selection of suitable patients for chemotherapy or immunotherapy. The present study deciphered that C1 and C3 were more sensitive to bortezomib and gemcitabine, respectively. The combination of the standard chemotherapy drug, temozolomide, and these two drugs can achieve better treatment in gliomas (Bastiancich et al., 2018; Kong et al., 2018). Further, according to the biological function and immune pattern analysis, we observed C1 and C3 belonged to the “immune-hot” subtype, and they also exhibited high expression of immune checkpoint molecules, which were the promising targets of immunotherapy. Thus, we suspected that C1 and C3 may be more sensitive to immunotherapy. Meanwhile, the results of TIDE and subclass mapping analyses evidenced our speculation. C1 was more sensitive to anti-PD1 therapy and ACT, and C3 was more sensitive to anti-CTLA4 therapy. Consistent with this, PD-L1 and CTLA4 were up-regulated in C1 and C3, respectively. In addition, combining the four clusters and clinical features, we developed an excellent nomogram for prognostic evaluation, which could guide more effective clinical management.

The present study also has some limitations. First, candidate genomic carcinogenic drivers and abundance of TME cells require further experimental verification. Second, patients suitable for bortezomib and gemcitabine have been predicted by bioinformatics algorithms, but further clinical validation is also required. Finally, intra-tumor heterogeneity was not considered due to the lack of relevant data.

## Conclusions

The present study revealed four heterogeneous glioma clusters with distinct genomic variants, functional phenotype, immune escape mechanism, and clinical characteristics. The nomogram with excellent performance was developed to serve as a powerful prognostic predictor. These results could enhance the mastery of molecular features and promote the precise therapy and clinical management of glioma.

## Data Availability

The datasets presented in this study can be found in online repositories. The names of the repository/repositories and accession number(s) can be found in the article/Supplementary Material.
