# Supplementary figures and images for: Comprehensive Molecular Analyses of a Novel Mutational Signature Classification System with Regard to Prognosis, Genomic Alterations, and Immune Landscape in Glioma
Source: Front Mol Biosci. 2021 Jul 7;8:682084. doi: 10.3389/fmolb.2021.682084 (PMC8293748; doi:10.3389/fmolb.2021.682084)

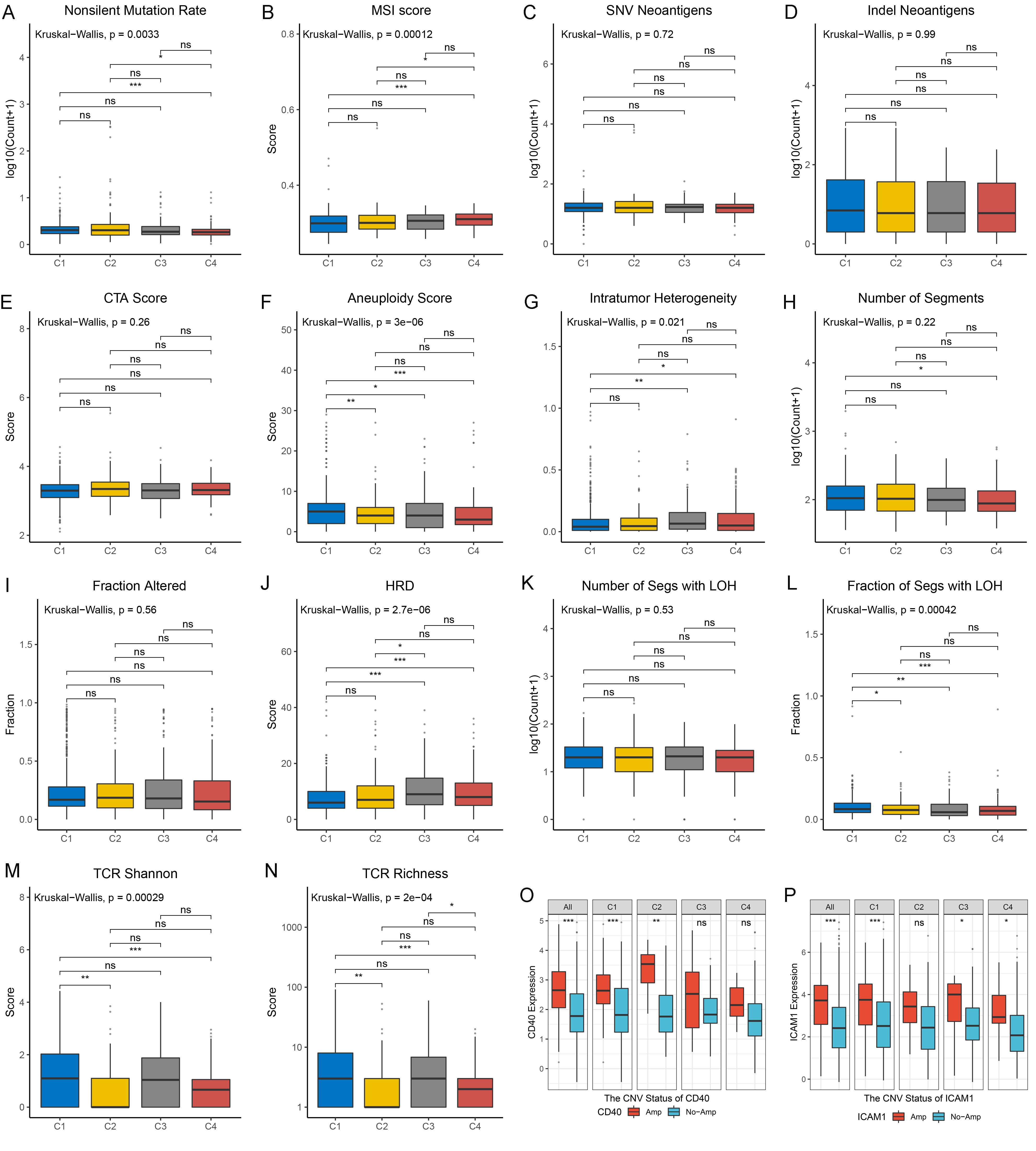

Supplement: Supplementary file 1 [file Image6.TIF]

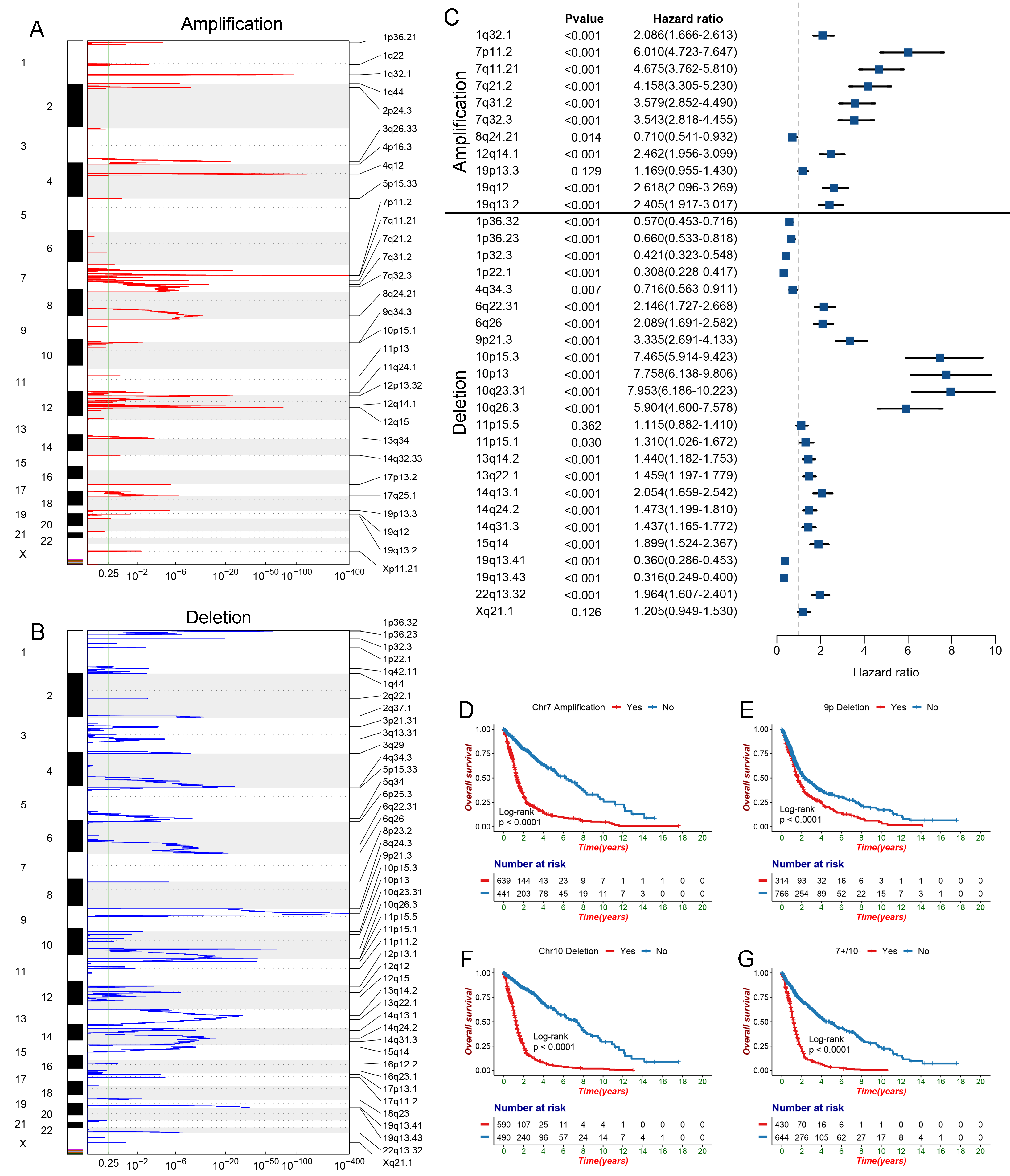

Supplement: Supplementary file 2 [file Image3.TIF]

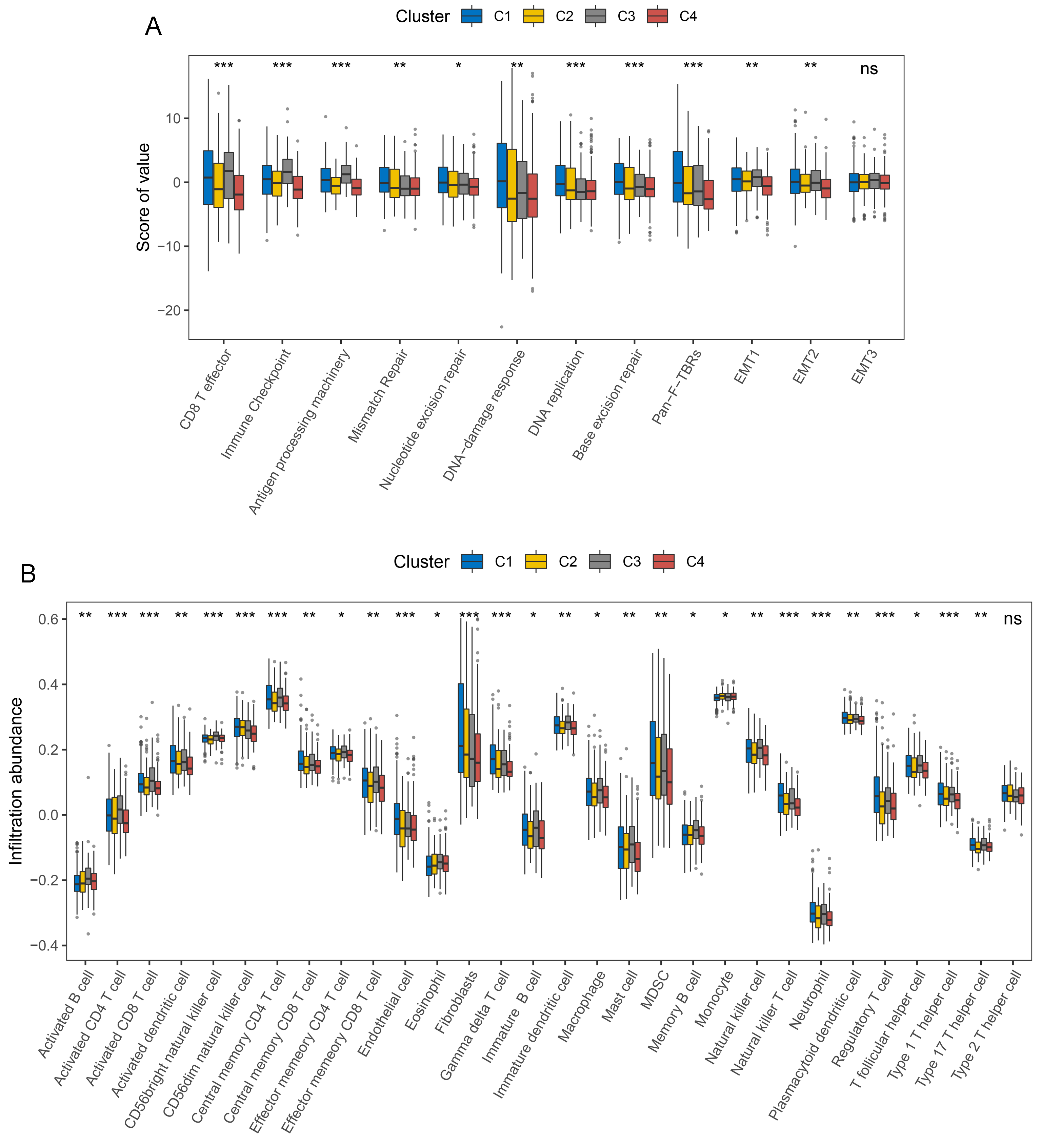

Supplement: Supplementary file 3 [file Image4.TIF]

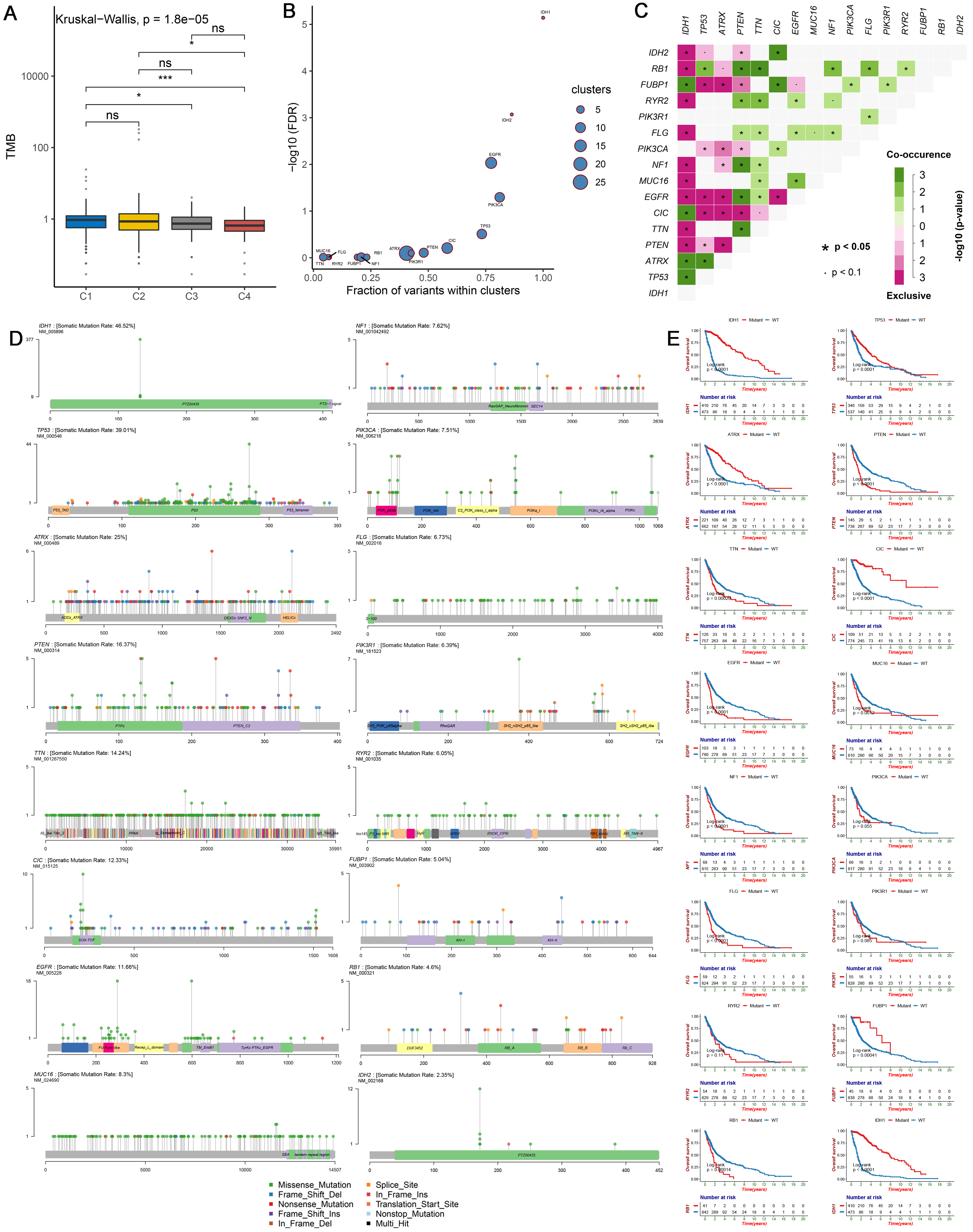

Supplement: Supplementary file 4 [file Image2.TIF]

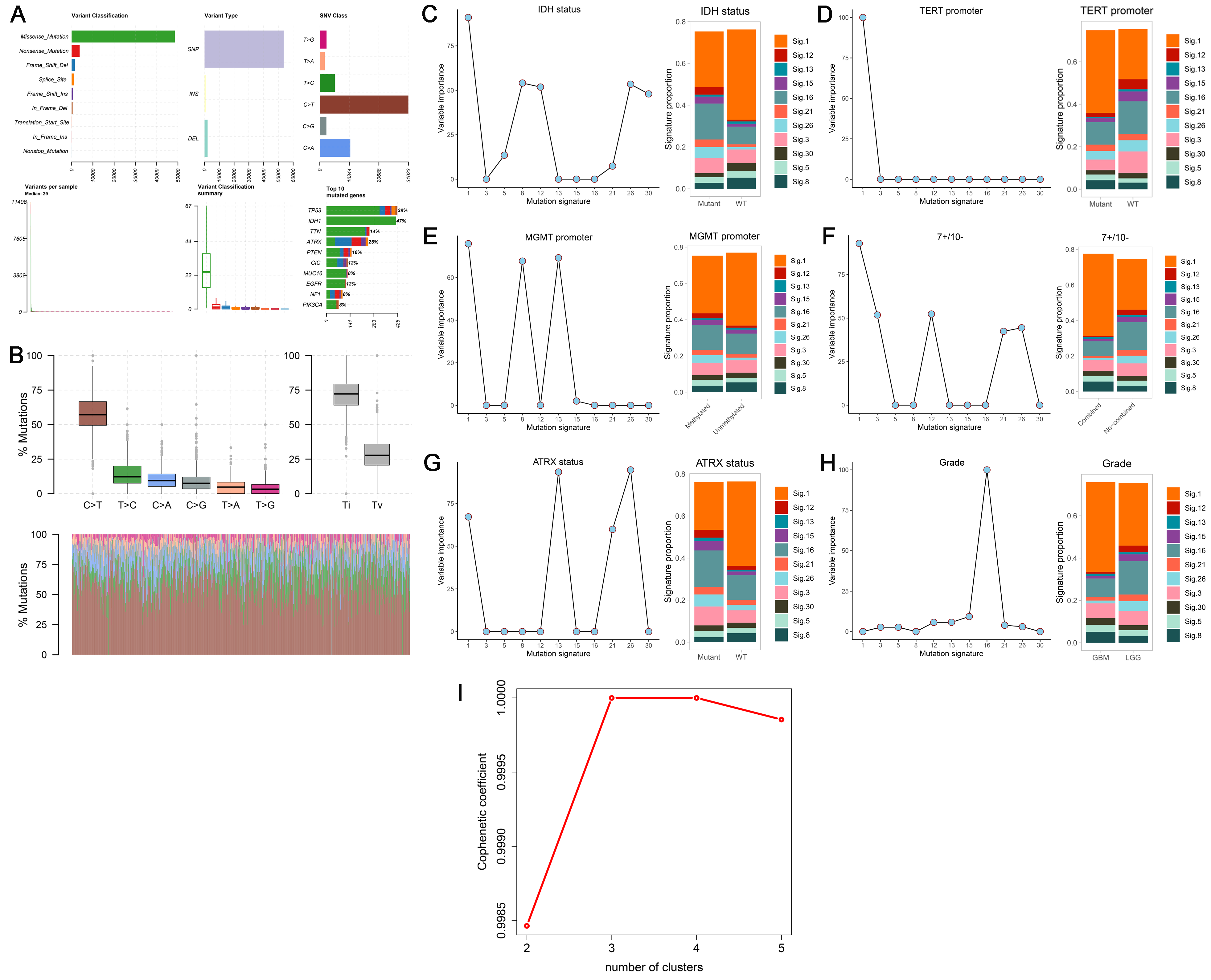

Supplement: Supplementary file 5 [file Image1.TIF]

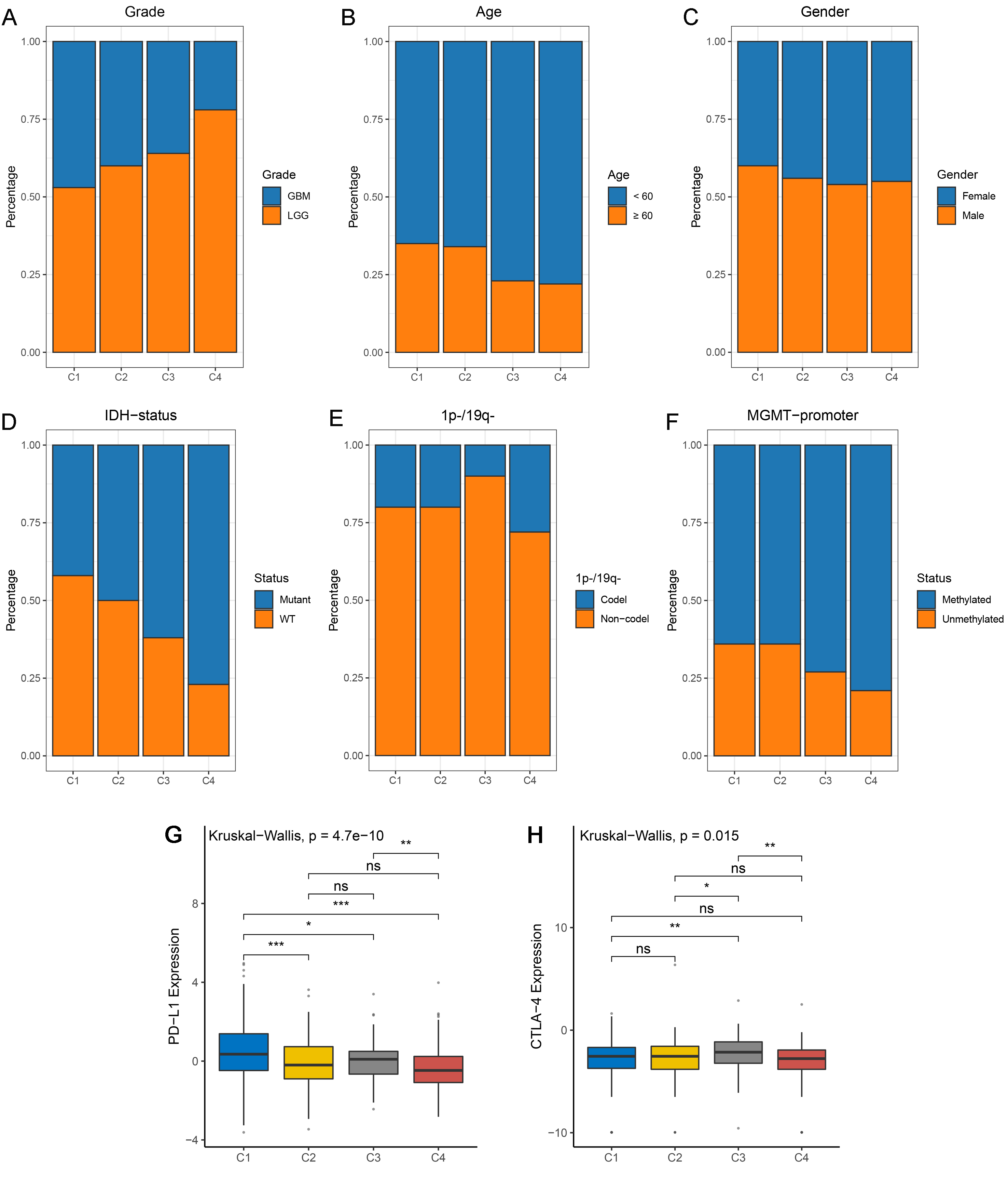

Supplement: Supplementary file 6 [file Image7.TIF]

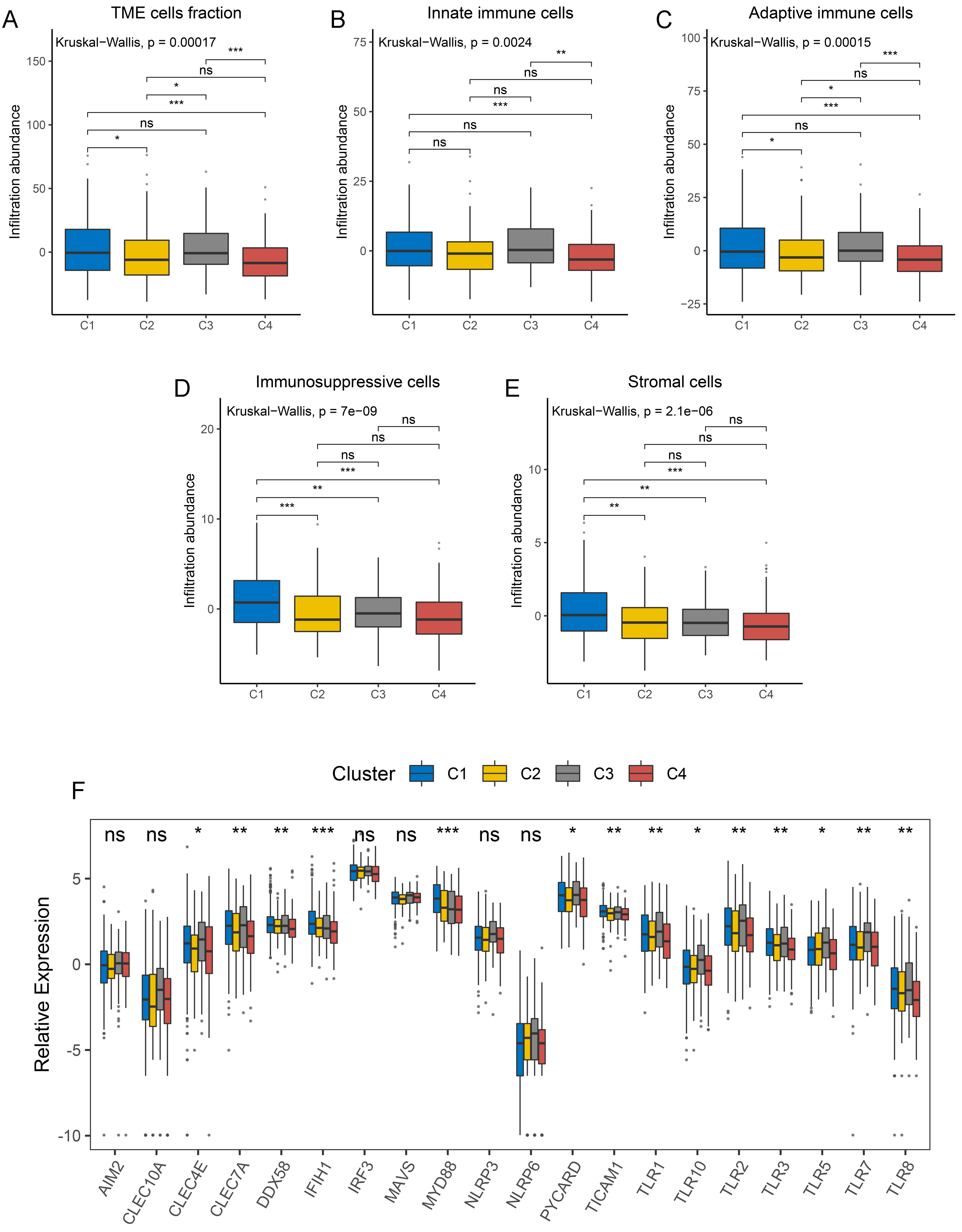

Supplement: Supplementary file 7 [file Image5.TIF]
